# The Role of MicroRNA as Clinical Biomarkers for Breast Cancer Surgery and Treatment

**DOI:** 10.3390/ijms22158290

**Published:** 2021-08-01

**Authors:** Matthew G. Davey, Molly Davies, Aoife J. Lowery, Nicola Miller, Michael J. Kerin

**Affiliations:** Department of Surgery, The Lambe Institute for Translational Research, National University of Ireland, Galway, H91 YR71 Galway, Ireland; m.davies1@nuigalway.ie (M.D.); aoife.lowery@nuigalway.ie (A.J.L.); nicola.miller@nuigalway.ie (N.M.); michael.kerin@nuigalway.ie (M.J.K.)

**Keywords:** breast cancer, miRNA, non-coding RNA, precision oncology, personalised medicine

## Abstract

Breast cancer is the most common cancer diagnosed in women. In recent times, survival outcomes have improved dramatically in accordance with our enhanced understanding of the molecular processes driving breast cancer proliferation and development. Refined surgical approaches, combined with novel and targeted treatment options, have aided the personalisation of breast cancer patient care. Despite this, some patients will unfortunately succumb to the disease. In recent times, translational research efforts have been focused on identifying novel biomarkers capable of informing patient outcome; microRNAs (miRNAs) are small non-coding molecules, which regulate gene expression at a post-transcriptional level. Aberrant miRNA expression profiles have been observed in cancer proliferation and development. The measurement and correlation of miRNA expression levels with oncological outcomes such as response to current conventional therapies, and disease recurrence are being investigated. Herein, we outline the clinical utility of miRNA expression profiles in informing breast cancer prognosis, predicting response to treatment strategies as well as their potential as therapeutic targets to enhance treatment modalities in the era of precision oncology.

## 1. Clinical Breast Cancer: Tumour Heterogeneity and Precision Oncology

Breast cancer is the most common cancer in women, with estimations suggesting 1.67 million women are diagnosed and treated for new breast cancers each year [1]. Despite the increase in breast cancer incidence and the disease now being recognised as the second most common cause of cancer death in female patients, significant progress has been made in breast cancer patient management, with anticipated 5-year survival rates improved from 40% to 87% over the past five decades [2]. Our enhanced understanding of the biological processes driving the disease and the increasing discovery of effective treatment options have resulted in a decrease in breast cancer mortality of 2–3% per year in the developed world [3]. While complete surgical resection remains the cornerstone of breast cancer control, recent advances in treatment options have facilitated more refined and personalised approach to breast cancer patient care. These timely enhancements of breast cancer care coincide with our heightened appreciation for molecular, cellular, and genomic properties driving oncogenesis in the molecular era. We now recognise a novel taxonomy of breast cancer which classifies four distinct clinically relevant molecular subtypes, i.e., Luminal A breast cancer (LABC), Luminal B breast cancer (LBBC), human epidermal growth factor Receptor-2-enriched breast cancer (HER2) and basal-like triple-negative breast cancer (TNBC) [4]. The gold standard in classifying breast tumours into these intrinsic biological subtypes is determined using multigene signatures (such as PAM50 assay from NanoString Technologies, Seattle, Washington, USA). However, the routine immunohistochemical appraisal of the estrogen (ER), progesterone (PgR) and HER2 receptor, as well as proliferation indices (Ki-67) in locally accredited histopathology laboratories, are also utilised in practice [5].

Despite our efforts to substratify cancers into prognostic subgroups, tumour behaviour and prognosis remains unpredictable and adds difficulty in attempts to optimise strategies to improve disease control while minimising toxicities to patients. Precision oncology relies on strategies such as genomic profiling to personalise care for breast cancer patients; the 21-gene expression assay (OncotypeDX Recurrence Score©, Genomic Health Inc., Redwood City, CA, USA) is routinely used in ER+/HER2-node-negative early breast cancer patients to select those who will derive the most benefit from systemic chemotherapy prescription, with first results from trial data supporting the expansion of indications to include those with 1–3 positive axillary nodes [6]. Within hereditary breast cancer, genetic profiling is used to identify patients with BRCA1/2 mutations to determine strategies surrounding prophylactic mastectomy. Furthermore, breast oncology has progressed in recent years to recognise the inherent value of treating patients with chemotherapy in the neoadjuvant setting. Advantages such as tumour downstaging and increased breast conserving surgery are beneficial for patients hoping to avoid mastectomy [7,8]. Moreover, the neoadjuvant prescription of systemic therapies allows for the generation of in-vivo data in relation to tumour sensitivity, which has been illustrated to carry prognostic significance for disease recurrence and survival. These modern facets of conventional breast cancer management provide insight into the potential utility of novel biomarkers in enhancing the current treatment paradigm. At present, there is a paucity of biomarkers capable of accurately predicting response and resistance to current systemic and targeted therapies, while efforts to employ non-invasive techniques to capture such biomarkers have proven futile to some extent. This reinforces the high priority for scientists to detect novel biomarkers capable of detecting response to treatment, inform the prognosis of patients diagnosed with breast cancer and provide clinicians with novel therapeutic strategies to target oncogenesis. This review focuses on the role of microRNA (miRNA) as emerging clinical biomarkers within the context of breast cancer surgery and treatment.

## 2. miRNAs as Breast Cancer Biomarkers

miRNAs are small (19–25 nucleotides in length), endogenous, non-coding RNA understood to play important regulatory roles in governing gene expression and cellular activity. Aberrant miRNA expression profiles have been observed in a diversity of pathological processes, including cancer development [9]. miRNAs have been demonstrated to regulate gene expression at a post-transcriptional level via binding to 3′ or 5′ untranslated regions of target messenger RNA (mRNA), directly impairing the mRNA degradation or inhibition of translation. In addition to their inhibitory role, miRNAs have been described to facilitate increase in transcript levels, increasing gene expression in certain circumstances [10].

First described by Lee et al. in 1993 [11], miRNA expression has been critically implicated in the development of human cancers, with translational research efforts growing exponentially in recent years [12,13]. miRNA biogenesis is a complex, multistep processes which is initiated in the cellular nucleus, where miRNA genes undergo transcription by RNA polymerase II/III to form large capped and polyadenylated primary miRNA transcripts (pri-miRNAs). The cleavage of these molecules by the coupled RNase III enzyme Drosha and its complementary binding partner DCGR8 produce pre-miRNA (70–90 nucleotides in length). These pre-miRNAs are the precursors to miRNA and are transported out of the cellular nucleus by the export protein Exportin 5, in their “imperfect” hairpin structures [14]. In the cytoplasm, these pre-miRNAs are cleaved by RNase type III Dicer with either the trans-activating RNA-binding protein (TRBP) or the protein activator of the interferon-induced protein kinase (PACT) [15], with one strand of this miRNA duplex representing mature miRNA which forms the RNA-induced silencing complex with other proteins [16]. This mature strand is preferentially incorporated into the miRNA-associated RNA-induced silencing complex (miRISC), which guides the RISC to target mRNA with complementary sequences to the mature miRNA. This ultimate step is responsible for impacting cellular activity.

miRNAs may be used to further substratify breast cancers into distinct subtypes, and miRNA expression profiles have successfully been utilised to predict steroid hormone receptor status [17], implicating targeting such biomarkers may be an advantageous strategy in dysregulated-receptor-associated miRNAs. Aberrant miRNA expression has been correlated to epithelial–mesenchymal transition (EMT), highlighting their critical role in cancer pathways capable of inducing distant metastasis [18,19]. These are examples of the crucial role of miRNAs within the breast cancer paradigm and are vital for the clinical scientists in the efforts to develop novel therapeutic strategies to enhance patient outcomes and inform prognoses. Data supporting miRNAs as modulators of genetic expression within breast cancer place them as obvious candidate prognostic and diagnostic biomarkers [20], as well as potential therapeutic targets. Furthermore, the unique capability of these molecules to maintain their stability over prolonged time makes them favourable informative biological parameters [21]. However, there remains limitations of miRNAs as biomarkers in breast cancer: At present, the absolute quantification of miRNAs following quantitative real-time polymerase chain reactions provides challenging to the current translational research effort, with inconsistencies observed in results limiting their implementation into clinical practice. The quantification of miRNAs in liquid biopsy form also yields inconsistent results, with apparent uncertainty being cast over what the optimal medium is (i.e., serum, plasma or whole blood) to evaluate miRNA expression levels [22]. These physical properties add further complexity to the routine implementation of miRNAs as biomarkers in the clinical setting of breast cancer workup and diagnosis.

## 3. MicroRNAs in Predicting Response and Resistance to Neoadjuvant Therapies

Oncological outcomes are enhanced by systemic chemotherapy prescription prior to or following cancer surgery, in particular when a multimodal therapeutic approach including endocrine agents, targeted therapies and radiotherapy are utilised [23]. The first chemotherapeutical regimen for operable breast cancer was prescribed in 1976 by Bonadanna et al. [24], where cyclophosphamide, methotrexate and 5-fluorouracil (CMF) significantly reduced breast cancer recurrence rates in 207 breast cancer patients compared to in controls (recurrence: 5.3% vs. 24.0%). Bernard Fisher and the National Surgical Adjuvant Breast and Bowel Projects (NSABP) first investigated the concept of systemic chemotherapy to enhance clinical outcomes in breast cancer through prospective, randomised control trials (RCTs): The seminal NSABP-B18 RCT involved the randomisation of over 1500 women to receive neoadjuvant or adjuvant doxorubicin and cyclophosphamide and was the original study investigating systemic chemotherapy prescription in the neoadjuvant setting [25]. Although results from NSABP-B18 (and more recent meta-analysis of RCTs) outlined no survival advantage for chemotherapy prescription in the neoadjuvant or adjuvant settings [26], an increase in the number of cancers amenable to breast conservation surgery (BCS) [25] was observed following a neoadjuvant approach. Since then, the focus has been adjusted to predict response rates to neoadjuvant chemotherapies (NAC) with efforts centred around predicting those likely to achieve favourable responses, such as pathological complete response (pCR), defined as complete eradication of the tumour from the breast and/or axilla, or those which are likely to develop resistance to treatment. Traditional molecular biomarkers such as ER, PgR, Ki-67 indices and Nottingham grade have all been used as predictive biomarkers of response to NAC. In more recent times, miRNA profiling has proven useful in dichotomising patients into those unlikely to response and those likely to achieve partial response or complete response to NAC (Table 1) [27,28,29,30,31,32,33,34,35,36,37]. Furthermore, the real-time monitoring of miRNA expression levels has the potential to enhance the sensitivity of current systemic therapies on the tumour or identify cancers likely to be resistant to treatment. Kolacinska et al. demonstrate the ability of miRNA panels to predict the response of basal carcinoma to NAC, with increased expression levels of miR-200b-3p and miR-190a differentiating good from poor responses [37], as did decreased expression of miR-512-5p. Bockhorn et al. describe the chemoresistant role of miR-30c through the regulation of the twinfilin1 actin-binding protein, a known promoter of EMT. MiR-30a inversely correlates with interleukin-11 expression in breast cancer, with low interleukin-11 correlating with relapse-free survival [38]. 

In their analysis of the blood serum of 56 breast cancer patients, Wang et al. illustrate reduced miR-125b levels to correlate with resistance to four cycles of neoadjuvant 5-fluorouracil, epirubicin and cyclophosphamide (FEC) [39]. Chen et al. describe the downregulation of miR-200c to correlate clinically with drug resistance in 39 breast cancer patients in receipt of 2–6 cycles of epirubicin with or without docetaxel [40], which were validated subsequently through the work of Kopp et al. [41]. Within the context of HER2-positive breast cancer, Jung et al. describe increased miR-210 levels in patients with residual disease following treatment with trastuzumab-based NAC [42], indicating chemoresistance. Zhao et al. present results from 93 breast cancer patients and 32 “healthy” controls outlining the predictive value of miR-221 in identifying patients likely to develop chemoresistance to NAC [32]. Such studies provide clinical relevance in identifying patients with large, locally advanced disease who are likely to respond to neoadjuvant therapies and facilitate BCS by proxy through tumour downstaging.

As described, in vitro studies have successfully identified miRNA likely to inform treatment response, while real-world data from the translational arms of the prospective, multicentre translational Neoadjuvant Lapatinib and/or Trastuzumab Treatment Optimization [NeoALLTO] trial and Clinical Trials Ireland All-Ireland Cooperative Oncology Research Group [CTRIAL-IE ICORG] 10/11 trial clinicals highlight the significance of circulating biomarkers to indicate response to neoadjuvant therapies (Table 2) [42,43,44,45,46,47,48,49,50,51,52]. In the NeoALLTO trial, an analysis of miRNAs as circulating biomarkers in 451 female patients was conducted, with 30 and 6 miRNA signatures developed to predict pCR at baseline and after 2 weeks of neoadjuvant treatment, respectively [43]. Of these, four miRNAs were validated in predicting response to neoadjuvant therapies. In trials similar to the aforementioned studies, pCR has become incorporated as a primary analytical endpoint in the next generation of prospective, neoadjuvant clinical trials. This is rationalised by the novel prognostic significance correlated with response to therapy within the landscape of breast cancer patient outcomes, with patients achieving pCR experiencing enhanced survival when compared to their counterparts with residual disease.

As outlined, miRNA profiling has proven a useful avenue to predict response and resistance to chemotherapy and other treatment modalities. Several studies suggest the reintroduction of specific miRNAs which are known to be downregulated during oncogenesis into cancer cells, in order to halt tumour growth and progression [53,54]. This hypothesis has the potential to provide therapeutic benefits; the restoration of the cells natural endogenous complement of miRNA may be achieved through the implantation of short synthetic duplex RNAs using the RISC or by inducing the genetic expression of the stem-loop pre-miRNA through extracellular vesicles. On the contrary, an alternative approach involves the utility of miRNA modulation to enhance sensitivity to current conventional therapeutic strategies; Miller at al. illustrate the role of miR-221/miR-222 overexpression in inducing tamoxifen resistance in HER2/neu-positive 4-hydroxytamoxifen-resistant (OHT^R^) breast cancer cell lines [55]. These effects of tamoxifen sensitivity were shown to be mediated by the direct target of miR-221 and miR-222, the cell cycle inhibitor p27^Kip1^. The authors manipulated levels of p27^Kip1^, which re-sensitised the cells to tamoxifen therapy, thereby highlighting the role in miR-221/miR-222 antagonism in cases of luminal breast cancer displaying resistance to endocrine agents.

Novel hypotheses surrounding the development of therapeutic and diagnostic strategies within breast oncology include the manipulation of heat shock proteins (HSPs), which play crucial roles in post-translational activities, in order to enhance drug delivery. Ozgur et al. have previously demonstrated that two miRNAs (miR-29a and miR-193b) are both associated with cancer through their contact with heat shock protein 70 (HSP70) [56], which provides potential to enhance treatment effects. The oncogenic role of miR-21 in cancer is well described [57,58], and Si et al. have assessed the utility of anti-miR-21 2-O-methyl or locked nucleic acid oligonucleotides for therapeutic targeting to inactivate the oncogenic impact of this “oncomiR” [59]. If combined with current conventional therapeutic strategies, these pre-clinical studies provide promise for miRNA targets to enhance cancer patient care, by reducing oncogenesis through manipulation of oncogenic miRNA expression patterns. Turning focus to the clinical setting, the seminal work of McGuire et al. in the CTRIAL-IE ICORG 10/11 prospective, multicentre translational trial highlights the value of miR-21 expression as a correlate to response to standard NAC in their analysis of 114 breast cancer patients [44]. Other studies evaluating the role of miRNAs to indicate treatment response has shown some promising results (as outlined in Table 1 and Table 2): Jung et al. implicate miR-210 as a predictive biomarker of response to trastzumab in HER2-positive breast cancer patients [42], with upregulation being associated with resistance to such therapies, while Ichikawa et al. also demonstrate the utility of miR-26a and miR-30b in mediating the impact of anti-HER2 therapies [60].

## 4. miRNA in Predicting Outcome in Operable Breast Cancer

Personalised breast cancer patient management is dependent upon a myriad of reliable predictive biomarkers capable of forecasting outcome. Traditionally, clinicopathogical variables such as age at diagnosis, disease burden and tumour grade provided insight into anticipated outcome and preoperative planning [61]. While the molecular era has shifted the paradigm toward encompassing intrinsic biological tumour parameters which inform treatment decisions and prognoses, the degree of disease burden remains paramount to preoperative surgical planning. The routine measurement of the ER, PgR, HER2 receptors and Ki-67 proliferation indices [62,63,64] furthers accurate prognostication through intrinsic molecular subtyping, with modern advances implicating features pertinent to the tumour microenvironment important in informing prognosis [65]. Several studies detail miRNA expression profiles in breast cancer tissue, outlining their importance in relation to nodal burden, disease recurrence and survival [58,66,67].

Although there are a limited number of studies correlating miRNAs with nodal status, Elango et al. provide a thorough report of a 40-miRNA panel capable of predicting lymph-node metastasis in breast cancer [68], with miR-205 and miR-214-3p also predicting overall survival (OS). These miRNAs could prove informative as a “double-sword” biomarkers useful for preoperative surgical planning and also acting to inform prognoses. Liu et al. describe miR-10b as a marker of distant disease recurrence in 195 patients initially naïve of nodal metastasis [69]. In their analysis of 159 breast cancer patients, Chen et al. created a novel 4-miRNA signature (miR-191-5p, miR-214-3p, miR-451a and miR-489), which is reliable in predicting lymph-node metastasis (area under curve (AUC): 0.932; OS (hazard ratio (HR)): 6.2; disease-free survival (DFS) (HR): 6.3) [70]. These promising results highlight the pertinence of miRNAs in breast cancer development and progression, with these four mi-RNAs working synergistically to act as a potential predictor of cancer metastasis and patient prognoses. Okuno et al. describe the relevance of combining typical clinicopathological data (i.e., tumour size and lymphovascular invasion) with miR-98 expression levels to predict sentinel lymph-node biopsy positivity in 100 ER+/HER2- breast cancer patients (AUC: 0.877) [71]. Although exploring the utility of miRNA expression profiles to inform preoperative surgical planning, data supporting miRNA expression in predicting survival outcomes are paramount in an attempt to personalise therapeutic strategies. Wang et al. highlight the critical role of miR-21 expression in promoting metastatic transformation in their analysis of 252 breast cancer patients [58], while Sporn et al. [67] link miR-9 expression levels with OS in 985 breast cancer patients in The Cancer Genome Atlas. Tokumaru et al. report a dual purpose of miR-143 increased expression through correlation with enhanced OS and also with the presence of favourable tumour microenvironment cells (macrophage-2 and T-helper-2 cells) in patients diagnosed with luminal breast cancer [72]. These results imply that the treatment of luminal cancers with immunomodulatory drugs may prove futile, as has been outlined in a recent meta-analysis [65]. In their cox regression analysis, Sheng et al. describe miR-4317 as a predictive biomarker of OS (HR: 2.108) [73], while Gao et al. provide log-rank Kaplan–Meier analyses to highlight the predictive value of miR-1, miR-4274 and miR-6880 (all *p* < 0.001) as biomarkers of survival in breast cancer. Moreover, Zhang et al. highlight the clinical relevance of increased miR-330 expression in predicting enhanced survival for breast cancer patients [49]. Table 3 outlines multi-miRNA signatures and their role in predicting outcome in breast cancer [74,75,76,77,78,79,80,81,82,83].

## 5. Limitations and Challenges of miRNAs as Biomarkers

Despite considerable funding, investment and resource distribution into the investigation of miRNA as reliable and reproducible clinical biomarkers in breast cancer research and treatment, we are yet to undercover novel biomarkers which can rival the principal ER, PgR, and HER2 receptors to inform breast cancer diagnosis, prognosis and therapeutic strategies. Since the emergence of the molecular era, genomic signatures such as the 21-gene assay and the Mammaprint© 70-gene assay (Agendia, Amsterdam, The Netherlands) has reliably and reproducibly informed prognoses, refined therapeutic systematic chemotherapy prescription and facilitated personalised cancer treatment in early-stage luminal diseases [84,85,86,87,88]. The identification and characterisation of miRNA expression which are as reliable and reproducible as these genomic panels limit current hypotheses, suggesting miRNAs may be impactful biomarkers in malignancy [89]. Biomarker signatures currently used in clinical practice, such as the aforementioned 21-gene and 70-gene assays, all rely on the absolute quantification of genetic targets from paraffin-embedded tumour specimens and are incredibly reproducible from patient to patient. In contrast, the diagnostic, prognostic and therapeutic utilisation of miRNAs is currently dependent upon relative quantification, thus imposing less consistent and translatable results. There are a number of additional inherent challenges observed in ensuring accuracy in miRNA measurement: There remain inconsistencies in consensus in relation to the preparation of miRNAs for evaluation, for example discrepancies in results in relation to the most appropriate medium from which miRNAs are extracted [90]. There are data suggesting that whole blood is a poor biological fluid as constituent cancer cells alter miRNA expression levels in circulation [90], and consensus in relation to plasma and serum has not been reached. Varying methodologies have been employed with respect to sample preparation, anticoagulation, centrifugation and storage properties, and polymerase chain reaction protocols have all contributed to interstudy variability and inconsistencies in reported outcomes [91,92,93]. The normalisation of miRNAs has proven problematic for scientists due to the lack of a universal consensus regarding an accepted, appropriate reference miRNA. McDermott et al. implicate miR-16 and miR-425 in combination as the primary endogenous (or “housekeeping”) reference targets for breast cancer [94]; however, data from Pritchard et al. imply miR-16 is imperfect in this role, as it is impacted by haemolysis [90]. Such conundrums add further inconsistencies to current research methods, limiting conclusions which may be drawn due to the creation of heterogenous results [90,95]. The translational research effort would greatly benefit from the standardisation of protocols in order to ensure the accurate comparability of results, which may be interpreted in a homogenous nature and translated into meaningful scientific results. This may be best achieved through the collaboration of an expert consensus panel to compile their views on the appropriate measures to improve the current practice surrounding miRNA measurement. Thus, the creation and implementation of a standardized protocol for miRNA measurements seems warranted, if these molecules are to be utilised routinely as prospective diagnostic or prognostic biomarkers in cancer patient care.

The retention of the overall stability of these biomarkers in circulation between timepoints and individuals [96] remains a challenge in miRNA therapeutics, particularly with variation in expression levels at different timepoints and between certain individuals [96]. Another primary challenge in cancer therapeutics is the successful delivery of miRNAs to the target tissue in cancer, and there is an increase in the enhanced permeability and retention (EPR) effect, which causes poor blood perfusion, leading to a reduction in the efficacy of the delivery of miRNAs to local tissues [97], impacting these biomarkers as reliable treatment options. The utilisation of liposomes to increase delivery of miRNA [98], the introduction of molecules to positively impact the EPR effect [99], as well as the use of delivery vehicles such as exosome-encapsulated miRNA delivered through mesenchymal stem cells [100,101] and viral vectors have been deployed to increase miRNA delivery [99] into target tissues. Ambitions to manipulate complex facets of miRNA delivery are pertinent currently; however, promising breakthroughs are awaited eagerly.

Lastly, simple host and environmental factors such as patient age, gender, smoking habits and local trauma may impact miRNA expression profiles [102,103,104,105]. Fundamentally, this limits conclusions which may be drawn in relation to miRNA as accurate biomarkers indicative of cancer-related outcome, particularly in the setting of small patient sample sizes in pre-clinical research studies facilitating the scrutiny of results relating to miRNA expression profiles. In such incidences, the complexity of miRNA expression requires more interrogation than simple correlation with variable clinicopathological data in the hope of deriving statistically significant results. Thus, the interrogation of the scientific method with robust data is warranted in further translational research studies evaluating the relevance of miRNAs in clinical breast cancer management.

## 6. Future Directions

The correlation between aberrant miRNA expression patterns within tumourgenesis and disease development illustrates the hypothesis fuelling efforts to use miRNAs targeting to discover the next generation of anti-cancer therapeutics. As previously outlined, novel hypotheses and relevant therapeutic and diagnostic strategies include the alteration of HSP function, potential manipulation of “oncomiR” expression through the addition of 2-O-methyl or locked nucleic acid oligonucleotides for the therapeutic inactivation of the oncogenic impact of these targets, as well as other novel strategies to enhance tumour suppressors or reduce oncogenic miRNA expression patterns. Future directions for the next generation of prospective, translational research studies may be built on the previous scientific escapades of these previous authors to better inform patient prognostication, develop novel therapeutic strategies which utilise miRNAs as potential targets and ensure miRNA appraisal is focused at enhancing treatment effects and the improvement of clinical outcomes for those who succumb to new breast cancer diagnoses or recurrence.

## Figures and Tables

**Table 1:** Studies correlating microRNA (miRNA) expression profiles to response to neoadjuvant chemotherapy.

Author	Year	Country	Tissue	N	LOE	Neoadjuvant Treatment	miRNA Expression
Liu [27]	2017	China	Serum	86	N/R	EC & DTX	At the end of C2, reduced miR-34a correlated to response to NAC.
Ohzawa [28]	2017	Japan	Tumour tissue	47	Retrospective (III)	Anthracycline, DTX & Trastuzumab	The decreased expression of 13 miRNA predicted pCR and the increased expression of 4 miRNA-predicted pCR in HER2+ disease.
Garcia-Vazquez [29]	2019	Mexico	Tumour tissue	35	Retrospective (III)	5-FU, cisplatin & PTX	Low miR-143 predicted pCR in TNBC patients.
Garcia-Garcia [30]	2019	Mexico	Tumour tissue	32	Retrospective (III)	N/R	MiR-145-5p expression is associated with pCR in TNBC.
De Mattos-Arruda [31]	2015	Spain	Tumour tissue	52	Retrospective (III)	Anthracycline, DTX & Trastuzumab	Increased miR-21 expression levels correlated to response to treatment in HER2+ cancers.
Zhao [32]	2011	China	Plasma	27	Retrospective (III)	Epirubicin, DTX or PTX	Increased miR-221 expression levels predicted poor response to neoadjuvant therapies.
Raychaudhuri [33]	2017	Germany	Tumour tissue	64	Retrospective (III)	Epirubicin & PTX or EC & DTX	High miR-7 and reduced mir340 expression levels predicted response to NAC.
Liu [34]	2019	China	Serum	83	Retrospective (III)	DTX, Paraplatin & Trastuzumab	Decreased miR-21 expression levels in serum associated with clinical response to NAC
Liu [35]	2017	China	Serum	118	Retrospective (III)	EC & DTX	Serum measurements of miR-21 and miR-125b predicted response to NAC (combined AUC: 0.958)
Chekhun [36]	2020	Ukraine	Serum	182	Retrospective (III)	5-FU, DXR & cyclophosphamide or DXR & cyclophosphamide	Aberrant levels of miR-21, miR-182 and miR-205 predicted response to NAC in Luminal A breast cancer.
Kolacinska [37]	2014	Poland	Tumour tissue	11	Retrospective (III)	Various	Increased miR-190a, miR-200b-3p and miR-512-5p expression levels predicted pCR in TNBC.

N, number; LOE, level of evidence; NAC, neoadjuvant chemotherapy; C2, cycle 2; N/R, not reported; EC, epirubicin and cyclophosphamide; DTX, docetaxel; PTX, paclitaxel; 5FU, 5-fluorouracil; DXR, doxorubicin; TNBC, triple-negative breast cancer; HER2, human epidermal growth factor receptor-2; AUC, area under the curve.

**Table 2:** Prospective clinical studies correlating miRNA expression profiles to response to neoadjuvant chemotherapy.

Author	Year	Country	Tissue	N	LOE	Neoadjuvant Treatment	miRNA Expression
Di Cosimo [43]	2019	Italy	Plasma	429	Prospective (II); NeoALLTO trial (NCT: 00553358)	Trastuzumab, lapatinib & paclitaxel	Increased miR-140a-5p, miR-148a-3p and 374a-5p associated with pCR.
McGuire [44]	2020	Ireland	Whole blood	114	Prospective (II); Clinical Trials Ireland All-Ireland Cooperative Oncology Research Group [CTRIAL-IE ICORG] 10/11 (NCT: 00553358)	Various	Responders had reduced miR-21 and miR-195 vs. non-responders in all breast cancer subtypes. miR-21 predicted response (OR: 0.538; 95% CI: 0.308–0.943).
Jung [42]	2012	US/Korea	Plasma	72	Prospective (II)	5-FU, EC & trastuzumab	Lower miR-210 expression levels predicted pCR in HER2+ cancers.
Muller [45]	2014	Germany	Serum	127	Prospective (II); Geparquinto Trial (NCT: 00567554)	NAC with trastuzumab or lapatinib	miR-21, miR-210 and miR-373 were elevated in responders’ post-NAC for HER2+ cancers.
Al-Khanbashi [46]	2016	Oman	Tumour, TAN and serum	36	Prospective (II)	DXR, cyclophosphamide & DTX	Serum miR-451 expression levels decreased during NAC in clinical responders.
Rodríguez-Martínez [47]	2019	Spain	Whole blood	53	Prospective (II)	Various	miR-21 expression levels during NAC discriminated pCR, PR and SD.
Stevic [48]	2016	Germany	Plasma	211	Prospective (II); GeparSixto Trial (NCT: 01426880)	DTX or PTX +/- Carboplatin	Aberrant miR-199a associated with pCR to NAC
Zhang [49]	2020	China	Blood	65	Prospective (II); SHPD001 (NCT:02199418) & SHPH02 (NCT: 02221999)	PTX, cisplatin & trastuzumab	Low miR-222-3p expression levels predicted those achieving pCR (OR: 0.258; 95% CI: 0.070–0.958)
Kahraman [50]	2018	Germany	Blood	21	Prospective (II); Molecular DEtection of Breast cancer (MODE-B) study	Carboplatin & PTX	Mutli-miRNA panels predicted responders from non-responders to NAC in TNBC.
Zhu [51]	2018	China	Blood	24	Prospective (II); NCT:02041338	Epirubicin & DTX	Reduced miR-34a was observed in non-responders to NAC compared to in responders.
Di Cosimo [52]	2020	Italy	Plasma	429	Prospective (II); NeoALLTO trial (NCT: 00553358)	Trastuzumab, lapatinib & PTX	Multiple miRNA expression profiles correlated to pCR to lapatinib, trastuzumab or dual anti-HER2 therapy.

N, number; LOE, level of evidence, NCT, national clinical trial identifier; TAN, tumour-associated normal; OR, odds ratio; CI, confidence interval; TNBC, triple-negative breast cancer; HER2, human epidermal growth factor receptor-2; EC, epirubicin and cyclophosphamide; 5FU, 5-fluorouracil; DTX, docetaxel; PTX, paclitaxel; DXR, doxorubicin; pCR, pathological complete response; PR, partial response; SD, stable disease; NAC, neoadjuvant chemotherapy.

**Table 3:** miRNA signatures and their roles in predicting outcome in breast cancer patients.

Author	Year	Country	Tissue	N	miRNA Expression Signatures
Lai [74]	2019	China	Tumour & TAN	1044	Six miRNA signatures (miR-147b, miR-549a, miR-4501, miR-4675, miR-6715a and miR-7974) predicted OS at 5 years (AUC: 0.789).
Hong [75]	2020	China	Tumour	111	Eight miRNA expression signatures (miR-139-5p, miR-10b-5p, miR-486-5p, miR-455-3p, miR-107, miR-146b-5p, miR-324-5p and miR-20a-5p) predicted relapse and prognosis in TNBC (AUC: 800).
Cheng [76]	2018	China	Tumour & TAN	1207	Three miRNA expression signatures (including miR-133a-2, miR-204 and miR-301b) independently predicted OS (HR: 1.638; 95% CI: 1.147–2.339).
Shi [77]	2018	China	Tumour	1098	Three multi-miRNA signatures including miR-16-2, miR-31 and miR-484 predicted OS (AUC: 690).
Andrade [78]	2020	Brazil	Tumour	185	Four miRNA expression panels (miR-221, miR-1305, miR-4708 and RMDN2) substratified TNBC patients into high- and low-risk groups and independently predicted OS (HR: 0.32; 95% CI: 0.11–0.91).
Wu [79]	2020	China	Tumour & TAN	199	Aberrant expression levels of three miRNA (miR-21-3p, miR-200b-5p and miR-659-5p) independently predicted OS (HR: 7.396; 95% CI: 1.590–34.411).
Tang [80]	2019	China	Tumour	1098	Seventeen miRNA panels were constructed to predict OS, and a 13-miRNA signature predicted RFS.
Farina [81]	2017	US	Tumour	48	Six miRNA panels (miR-3124-5p, miR-1184, miR-4423-3p, miR-4529-5p, miR-7855-5p and miR-4446-3p), which predicted OS (AUC: 0.896; CI: 0.804–0.988).
Li [82]	2018	China	Serum	386	Four miRNA signatures (miR-16-5p, miR-17-3p, miR-451a and miR-940) predicted 1-year and 2-year predicted OS (AUC: 0.80 and 0.74, respectively) for metastatic HER2+ breast cancers.
Rohan [83]	2019	US	Tumour	530	Thirteen miRNA expression panels were designed to predict breast cancer recurrence (AUC: 0.67; CI: 0.58–0.795).

N, number; TAN, tumour-associated normal; OS, overall survival; AUC, area under the curve; TNBC, triple-negative breast cancer; HR, hazard ratio; CI, confidence interval; RFS, recurrence-free survival; US, United States; HER2, human epidermal growth factor receptor-2.

## Data Availability

Not applicable.
